# CAM Practices and Treatment Adherence Among Key Subpopulations of HIV+ Latinos Receiving Care in the San Diego-Tijuana Border Region: A Latent Class Analysis

**DOI:** 10.3389/fpubh.2019.00179

**Published:** 2019-06-28

**Authors:** Charles Marks, María Luisa Zúñiga

**Affiliations:** ^1^School of Social Work, San Diego State University, San Diego, CA, United States; ^2^SDSU-UCSD Joint Doctoral Program in Interdisciplinary Research on Substance Use, San Diego, CA, United States

**Keywords:** border health, complementary and alternative medicine, Latino health equity, HIV/AIDS, ART adherence

## Abstract

Latinos living in the United States-Mexico border region bear a disproportionate HIV/AIDS burden compared to individuals living in the interior of both nations and face a constellation of barriers that determine their ability to access and adhere to HIV care. Use of complementary and alternative medicine (CAM) may be associated with suboptimal treatment adherence. Sociodemographic factors, health practices, and social determinants of health unique to the border region may further contribute to health disparities that undermine care engagement and continuity. Improved understanding of HIV-positive Latino subgroups and their risk profiles can lead to more effective, targeted clinical and public health interventions. We undertook this study to identify and characterize distinct classes of HIV-positive Latinos in the San Diego-Tijuana border region, differentiated by HIV and border-related factors, utilizing latent class analysis. We investigated relationships between class membership and CAM utilization and self-reported antiretroviral therapy (ART) adherence. Five distinct classes were identified with unique demographic, HIV risk, and border mobility profiles. CAM was recently used by nearly half of each class, though there were significant differences in the proportion of CAM use by class ranging from 44.4 to 90.9%. As well, all classes were currently receiving ART at similarly high rates and ART adherence outcomes were not significantly different based on class. Findings highlight the significant use of CAM by all HIV-positive Latinos in the border region and imply the need for a research framework which appropriately acknowledges the heterogeneous nature of this population, such as intersectionality. Further research is recommended into understanding how patients integrate CAM into HIV treatment and the risks and benefits of incorporating CAM into HIV treatment.

## Introduction

In order to meet the United Nations goal of identifying, treating, and achieving viral suppression in 90% of all people living with HIV/AIDS (PLWHA) globally (1), it is necessary to understand the specific barriers and challenges faced by subpopulations that are disproportionately impacted by HIV. For Latinos in the United States (US) and Mexico, especially those residing in the shared border region, efforts to improve HIV-related health outcomes will require further attention to the unique needs of Latino subpopulations, especially those factors that place this vulnerable group at higher risk for falling out of care (2).

It is well-documented that US Latino immigrants face unique challenges to remain engaged in care throughout the continuum of HIV care (3–6). Yet, there is a need to better understand the barriers faced by specific Latino subpopulations in the US-Mexico border region where binational mobility is, for many, a part of daily life. The United States-Mexico Border Health Commission (USMBHC) identified slowing the spread of HIV in the border region as a primary objective by 2020, doing so after failing to meet their 2010 goal of significantly reducing the HIV incidence rate (7). In border cities such as Tijuana, studies have documented that HIV is concentrated within different high-risk communities, such as men who have sex with men (MSM), people who inject drugs (PWID), and female sex workers (FSW) (8). Though there is literature dedicated to understanding and addressing HIV-related health needs amongst MSM (9, 10), PWID (11–13), and FSW (14–17) in the US-Mexico border context, there is a need to understand the experiences of different subpopulations as they engage with health care services. While limited research into the attitudes of primary HIV care providers in the border region indicates that clinicians understand that there exist both personal and structural barriers to care for PLWHA (18), identifying which PLWHA populations are accessing care in the region remains an area of research need. Improved understanding of border patient profiles can inform clinical and health system efforts to promote care continuity and improved health outcomes.

The San Diego-Tijuana region provides an ideal setting for understanding the complex nature of living with HIV in the border region. As described by the USMBHC, the border region should not be conceptualized as two distinct neighboring nations, but as a set of independent twin cities, each of which represent environments requiring unique consideration (7). While comparisons of HIV rates between Tijuana and San Diego are difficult to ascertain due to different reporting standards in each country (19), HIV and AIDS disproportionately impact the region with higher rates than their respective national averages. In the state of Baja California, whose most populous city is Tijuana, the AIDS-related mortality rate is double the national average and this rate increased by nearly 20% from 2003 to 2013 (20). The HIV incidence rate for Latinos in San Diego County exceeds both the state and national general incidence rates (21, 22) and Latinos in the County went from representing 35% of new HIV cases in 2000 to 43% in 2014 (22). Further alarming is that 36% of all Latino PLWHA in San Diego County progress to AIDS within 30 days of their initial diagnosis, the highest rate amongst any race or ethnicity in the county (22). Overall, it is clear that much work is needed to achieve the United Nations 90% viral suppression goal, as overall in San Diego County in 2015 less than half of those diagnosed with HIV achieve viral suppression (22).

Characterizing PLWHA subpopulations in the San Diego-Tijuana region is not simply an exercise in identifying known high-risk communities, such as MSM and PWID, but must also account for the ways in which the border impacts individual mobility and access to care. The San Diego-Tijuana border is home to the busiest land border crossing in the western hemisphere where daily, tens-of-thousands of the region's citizens cross back and forth for work, family, shopping, school, and health care-related activities (23). Fluidity of dwelling (U.S. or Mexico or both) and binational mobility are hallmarks of living in the region (24). As the population of approximately 60 million Latinos in the US continues to grow by over 1 million people per year (25), achieving goals related to the HIV care cascade will require understanding of the barriers that Latino PLWHA face in accessing and remaining in HIV care. This includes understanding complex relationships between HIV-related risk factors, the impact of the border, as well as other relevant socio-demographic variables.

Additionally, the use of complementary and alternative medicine (CAM), such as herbal remedies or treatment from traditional healers, may play a complicating role in ensuring effective HIV care administration to PLWHA due both to inhibiting communication between patient and care provider (26, 27), as well as concerns about possible interactions between CAM and antiretroviral therapy (ART) (28). In the US, it is estimated that 32–55% of PLWHA utilize CAM to treat HIV-related symptoms (27, 29) and that rates of use are likely even higher amongst Latino PLWHA in the US-Mexico border region (26, 30). It appears, as well, that some Latino PLWHA utilize CAM when their care provider is unable to provide culturally appropriate treatment (31). Qualitative research has indicated that some HIV care providers in the San Diego-Tijuana region do not communicate with their patients about CAM, do not feel that they know enough about the interactions of CAM with ART medication to make recommendations, and have limited understanding of the role CAM use plays in ART adherence (32). For these reasons, it is important to understand CAM utilization among different Latino PLWHA subpopulations in San Diego and Tijuana to support clinicians in their efforts to have culturally-effective CAM dialogue with their patients.

One approach to understanding subpopulation profiles is the application of latent class analysis (LCA), a statistical tool that has been utilized to identify and characterize meaningful subpopulations, herein referred to as “classes.” Earlier studies have applied LCA to better understand subpopulation HIV risk profiles (33–35), with a primacy of literature focused specifically on class differences in HIV risk based on substance use behaviors (36–40). These studies have largely focused on characterizing substance using populations in both San Diego and Tijuana, identifying distinct classes of PWID with varying HIV risk profiles (36, 37, 40). Additionally, LCA has been utilized to identify PLWHA classes and predict their ART adherence (41, 42). In this study, we apply LCA to classify and characterize Latino sub-populations of PLWHA receiving HIV care in clinics in Tijuana and San Diego for the purpose of identifying present classes and to assess the barriers to adhering to and remaining in care the members of these classes face. Utilizing LCA, the primary objectives of this study are to: (1) identify and characterize classes of a population of PLWHA in San Diego-Tijuana based on HIV-risk factors and the impact of the border; (2) assess the relationship between class membership and CAM utilization; and (3) assess the relationship between class membership and care adherence.

## Methods

### Study Design

We conducted secondary data analysis using data from the cross-sectional study *Complementary and Alternative Care Behavior in HIV*+ *Latinos in the US-Mexico Region* (09/2009-07/2012), a mixed-methods study whose primary goal was to understand the relationship between CAM and factors associated with HIV care seeking and treatment adherence in the San Diego-Tijuana region. Participants were recruited simultaneously in San Diego and Tijuana from HIV health care and social services agencies. Trained interviewers administered a quantitative survey addressing CAM, ART, and other factors associated with HIV care seeking behavior in the U.S.-Mexico border region. The study adapted the Anderson and Aday Health Care Utilization model (43), which establishes a framework for integrating individual, structural, and provider-related factors in accessing healthcare service utilization. The study was approved by the UCSD Human Research Protection Program.

### Participants

The inclusion criteria for the study were that participants be Latino, diagnosed as HIV-antibody positive, having resided in San Diego or Tijuana for at least 1 month in the year prior, able to speak English or Spanish, be at least 18 years old, and be able to give informed consent. Participants were recruited from HIV care clinics and social services agencies in San Diego (*n* = 100) and Tijuana (*n* = 101).

### Measures

The survey was comprised of 11 sections based on existing scales and the author's prior work in binational care practices in the U.S.-Mexico border. The sections were as follows: *Demographics*, including age, education, language preference, living situation, and reported weekly income in US dollars (conversion rate of 12.5 Mexican pesos per 1 USD used based on 2009–2012 rates) (44); *Health History and Utilization of HIV Medical Services*, including health insurance status and recent medication history; *HIV Testing*, inquiring about HIV testing history, the progression from HIV positive test result to receiving care, as well as ART adherence; *Complementary and Alternative Medicine*, measuring CAM utilization and related attitudes [adapted from CAM domains defined by the National Center for Complementary and Alternative Medicine and on the California Health Interview Survey-Complementary and Alternative Medicine Questionnaire (45)]; *Religion/Spirituality*, asking about religious identification and role of spirituality in living with and managing HIV; *HIV-Related Felt Stigma Scale*, measuring HIV-related stigma utilizing measures validated for Spanish-speaking Latinos (46) and region-specific indicators defined in previous research (47, 48); *Knowledge, Attitudes, and Beliefs About HIV Care*, asking about attitudes around treatment adherence and CAM use as appropriate treatment modalities; *Border Crossing and Deportation*, asking about border crossing behaviors and related complications; *Sexual, Drug Use, and Other Risk Behaviors*, asking about history of substance use, sexual history, and other risk factors such as prior incarceration; *Mental Health/Depression*, utilizing the CES-D scale (49) to assess mental health status; and *Physician-Patient Relationship Quality Scales*, measuring elements of perceived relationship with primary HIV care provider based on HIV Cost and Services Utilization Study measures (50).

### Statistical Analyses

Our primary objective was to identify and characterize subpopulations of study participants. Latent class analysis (LCA) was utilized (RStudio v1.0.153, poLCA library) to assign individuals into classes based on the following dichotomous indicators: sexuality [heterosexual/Lesbian Gay Bisexual Transgender (LGBT)]; lifetime history of illicit drug use (never/ever); lifetime history of trading sex (never/ever); having health coverage in Mexico (yes/no); and having health coverage in the United States (yes/no). These were chosen to capture factors related to known HIV risk populations (MSM, PWID, and FSW) and to include border-related factors that are thought to be associated with HIV care access. Lifetime history of injection drug use was a preferred indicator, but due to low response rates (*n* = 138), lifetime history of illicit drug use was used in its place. As well, health coverage status was viewed to be a strong indicator for border impact because, (a) nearly all (*n* = 196) participants reported having health coverage and (b) it accounted for participant ability to access care on either side of the border, as opposed to only accounting for the location where the participant was surveyed. Missing indicator values were imputed as a part of the poLCA library's LCA implementation.

LCA models were fit to 2, 3, 4, 5, 6, and 7 classes and the Akaike's information criteria (AIC) and the Bayesian information criteria (BIC) were applied to assess and compare the goodness-of-fit of each of the models (51). To choose the best fit model, the AIC and BIC were considered in addition to a qualitative determination of which model provided the most clinically meaningful classification. The LCA assigns a probability that each participant is a member of each class. The participants were assigned to the class of the chosen model of which they had the highest probability of being a member. Then, descriptive statistics for each class were generated (tableone library) and were utilized to characterize each class. In addition, Chi-squared tests and ANOVA were applied to determine significant class differences on demographic, HIV-related risk, CAM utilization, and ART adherence factors.

## Results

Of 201 total participants, 69.3% reported residence in Mexico at the time of the interview, 82.4% were male, the mean age was 48 years (median 48.00), and half had not completed secondary school. The mean income (in US $) was $137.50/week though 17.9% reported having no income at all and 45.8% were unemployed. In the prior 6 months, 36.7% reported having made at least one round-trip border crossing. In total, 62.8% identified as LGBT, 58.7% reported lifetime use of illicit drugs (excluding cannabis), and 34.7% had traded sex. Twelve individuals, all men, reported having between 10 and 30 sex partners in the prior 3 months. In all, 71.1% had reported using CAM in the prior 6 months with teas (60%), vitamins (60%), herbs and plants (27.9%), and body work (23.4%) representing a majority of the reported use. While 72.5% reported being at least in “good” health, 40.1% had been diagnosed with AIDS at the time of their interview. Almost 90% were currently taking ART medication of whom 28.5% reported missing taking their medication at least once in the prior month. Of those who had been diagnosed over 3 months prior to being interviewed, 18.5% had at gone at least a 3-month span without taking ART medication.

### Latent Subpopulations

LCA fit statistics indicated that the 3-class and 5-class solutions presented the best fits (see Table 1), and here we present the results of the 5-class solution which was determined to provide the most clinically meaningful classification. First, we shall describe the classes individually based on the model indicators, demographic factors, and factors related to the border. For each such variable, there was a statistically significant difference across the five classes (see Table 2 to examine these differences and their significance values). Classes have been ordered by the proportion of the class which reported having health coverage in the US (highest to lowest) and are described here:

**Table 1:** LCA goodness-of-fit statistics^*^.

**Number of classes**	**AIC**	**BIC**
2	1245.262	1281.599
3	1211.834	**1267.991**
4	1208.335	1284.311
5	**1207.406**	1303.201
6	1218.18	1333.796
7	1221.817	1357.253

* *Best fit(s) indicated in bold*.

**Table 2:** Model indicators, demographics, border crossing, and HIV risk behaviors (by class).

	**Class**					
	**Total (*n* = 201)**	**1 (*n* = 27)**	**2 (*n* = 59)**	**3 (*n* = 48)**	**4 (*n* = 34)**	**5 (*n* = 33)**
**MODEL INDICATORS**						
Is LGBT? ^***^	125 (62.8%) ^∧^	16 (59.3%)	48 (81.4%)	46 (100%)	0 (0.0%)	15 (45.5%)
Lifetime, Used Illicit Drug, Except Cannabis^***^	118 (58.7%)	0 (0.0%)	59 (100%)	5 (10.4%)	21 (61.8%)	33 (100%)
Lifetime, Traded Sex^***^	68 (34.7%)	2 (8.0%)	29 (49.2%)	5 (10.4%)	0 (0.0%)	32 (100%)
Has Health Coverage in Mexico^***^	123 (61.2%)	0 (0.0%)	10 (16.9%)	46 (95.8%)	34 (100%)	33 (100%)
Has Health Coverage in United States^***^	106 (52.7%)	27 (100%)	56 (94.9%)	16 (33.3%)	7 (20.6%)	0 (0.0%)
**DEMOGRAPHICS**						
Age, Mean (SD)^**^	48 (9.6)	54.9 (14.2)	47.9 (7.6)	45.9 (8.52)	47.7 (8.8)	46 (7.9)
Sex, Male^***^	164 (82.4%)	24 (88.9%)	57 (96.6%)	45 (97.8%)	14 (44.1%)	23 (69.7%)
Country of Residence (Past 6 Months)^***^						
United States	53 (26.6%)	15 (57.7%)	34 (57.6%)	1 (2.1%)	2 (6.1%)	1 (3.0%)
Mexico	138 (69.3%)	7 (26.9%)	22 (37.3%)	46 (95.8%)	31 (93.9%)	32 (97.0%)
Both	8 (4.0%)	4 (15.4%)	3 (5.1%)	1 (2.1%)	0 (0.0%)	0 (0.0%)
Received Care in the US? (Study Location)^***^	100 (49.8%)	26 (96.3%)	53 (89.8%)	16 (33.3%)	5 (14.7%)	0 (0.0%)
Weekly Income US$, Mean (SD)^***^	137.5 (172.7)	215.5 (173.0)	200.7 (237)	138.2 (127.4)	63 (64.1)	32.9 (44)
Has No Income ^***^	36 (17.9%)	2 (7.4%)	7 (11.9%)	2 (4.2%)	8 (23.5%)	17 (51.5%)
Educational Attainment^***^						
Not Completed Secondary School (Grade 8)	100 (50.0%)	11 (40.7%)	29 (49.2%)	34 (72.3%)	12 (35.3%)	14 (42.4%)
Completed Secondary	20 (10.0%)	3 (11.1%)	12 (20.3%)	1 (2.1%)	3 (8.8%)	1 (3.0%)
Completed GED	24 (12.0%)	2 (7.4%)	2 (3.4%)	1 (2.1%)	7 (20.6%)	12 (36.4%)
Attended Post-GED Education	56 (28.0%)	11 (40.7%)	16 (27.1%)	11 (23.4%)	12 (35.3%)	6 (18.2%)
Lifetime, Ever Incarcerated^***^	62 (31.3%)	1 (3.8%)	12 (20.3%)	8 (17.0%)	14 (42.4%)	27 (81.8%)
Unemployed^***^	92 (45.8%)	14 (51.9%)	27 (46.4%)	9 (19.8%)	18 (54.5%)	24 (72.7%)
**BORDER CROSSING**						
Round Trip Border Crossing (Past 6 Months)^***^	73 (36.7%)	14 (53.8%)	34 (57.6%)	18 (37.5%)	6 (18.2%)	1 (3.0%)
Lifetime, Ever Deported From US to Mexico^**^	39 (20.3%)	2 (9.5%)	7 (12.1%)	7 (14.6%)	8 (24.2%)	15 (46.9%)
Lifetime, Ever Stopped by Border Patrol^*^	54 (28.4%	4 (16.0%)	11 (19.0%)	13 (29.5%)	10 (32.3%)	16 (50.0%)
**OTHER SEX AND DRUG BEHAVIOR**						
Number of Sex Partners (Prior 3 Months)						
Mean (SD)^***^	2.6 (5.1)	0.8 (0.9)	5.3 (7.7)	1.8 (3.1)	0.8 (1)	2.2 (4.2)
Used Condom Everytime (Prior Three Months)^***^	108 (74.0%)	14 (93.9%)	30 (58.8%)	34 (94.9%)	16 (80.0%)	14 (58.3%)
**Lifetime, Ever Used:**						
Cannabis^***^	110 (55.3%)	7 (26.9%)	48 (81.4%)	13 (27.1%)	12 (36.4%)	30 (90.9%)
Crack/Cocaine^***^	88 (44.2%)	0 (0.0%)	38 (64.4%)	4 (8.3%)	15 (45.5%)	31 (93.9%)
Methamphetamine^***^	50 (25.1%)	0 (0.0%)	25 (42.4%)	2 (4.2%)	7 (21.2%)	16 (48.5%)
Heroin^***^	33 (16.6%)	0 (0.0%)	10 (16.9%)	0 (0.0%)	7 (21.2%)	16 (48.5%)
Lifetime, Ever Injected Illicit Drug^*^	28 (20.3%)	0 (0.0%)	8 (13.6%)	1 (6.7%)	9 (37.5%)	10 (30.3%)

* *p < 0.05*,

** *p = 0.001*,

*** p < 0.001, and

∧ *proportions based on total respondents to survey items*.

#### Class 1 (*n* = 27)

In this class, every member was recruited in the US and all but 26.9% reported residing in the US over the prior 6 months. Every member reported having health coverage in the US and none in Mexico. Members were significantly older with a mean age of 55 years. In total, 88.9% were men and 59.3% of members identified as LGBT. This class had the highest mean income at $215.52/week and only 7.4% reported having no income despite 51.9% reporting not being employed, though this may be explained by retirement. Educational background was wide-ranging as 40.7% of individuals had not completed secondary school and the same proportion had received post-GED education. Only 8% reported ever having traded sex, members reported an average of 0.81 sexual partners in the prior 3 months, and 93.3% identified using a condom during sex every time in that same time span. Zero members reported lifetime illicit drug use or injection drug use. As well, only 3.8% reported ever having been incarcerated. In all, 53.8% had made a round-trip border crossing in the previous 6 months and 9.5% had ever been deported from the US to Mexico. Overall, this class can be characterized as older, wealthier individuals (potentially retired), primarily LGBT men, living in both Mexico and the US, receiving care in the US, with border mobility, with no history of using illicit drugs or trading sex.

#### Class 2 (*n* = 59)

In this class, 89.8% of members were recruited in the US, 62.7% had residence there in the prior 6 months, 94.9% had health coverage in the US and 16.9% had health coverage in Mexico. The mean age was 45.91 years. In total, 96.9% were men and 81.4% of members identified as LGBT. This class had the second highest mean income at $200.74/week and only 11.9% reported having no income despite 46.6% reporting not being employed. Approximately half, 49.2%, had not finished secondary school and 47.4% had completed high school. In total, 49.2% had ever traded sex, members reported an average of 5.29 sexual partners in the prior 3 months, skewed by multiple members reporting as many as 30, and 58.8% identified using a condom during sex every time in that same time span. Every member had a lifetime history of using illicit drugs, though only 13.6% had reported ever injecting. Just over 20% reported ever having been incarcerated. In all, 57.6% had made a round trip border crossing in the previous 6 months and 12.1% had ever been deported from the US to Mexico. Overall, this class can be characterized as men, primarily LGBT, living in both the US and Mexico, receiving care in the US, with a history of illicit drug use, with many members having traded sex, with a relatively higher income, border mobility, with lower likelihood of ever having been incarcerated.

#### Class 3 (*n* = 48)

In this class, 67.7% of members were recruited in Mexico, 95.8% lived there the prior 6 months, 95.8% had health coverage in Mexico and 33.3% had health coverage in the US. The mean age was 45.91 years. Men made up 97.8% of the class and every member identified as LGBT. Though the class average income was slightly less than the mean of the entire population at $138.17/week, this class had the lowest proportion of unemployed individuals at 19.8% and the lowest proportion of individuals with no income at 4.2%. Almost three-quarters of members had not completed secondary school. Only 10.4% reported ever having traded sex, members reported an average of 1.75 sexual partners in the prior 3 months, and 94.9% identified using a condom during sex every time in that same time span. In total, 10.4% reported lifetime us of illicit drugs, and of those that responded, 6.7% reported lifetime injection drug use. As well, 17% had ever been incarcerated. In all, 37.5% had made a round trip border crossing in the previous 6 months and 14.6% had ever been deported from the US to Mexico. Overall, this class can be characterized as LGBT men living in Mexico, likely working-class with low educational attainment, with minimal history of illicit drug use or sex work.

#### Class 4 (*n* = 34)

In this class, 85.3% of members were recruited in Mexico, 93.9% lived there the prior 6 months, and all group members had health coverage in Mexico. The mean age was 47.7 years. Men made up 44.1% of the class and every member identified as heterosexual. The mean income was the second lowest at $62.97/week, 23.5% reported no income at all, and 54.5% reported being unemployed. Educational background was wide ranging as 35.3% of individuals had not completed secondary school and the same proportion had received post-GED education. Zero members of the group reported ever having traded sex, members reported an average of 0.82 sexual partners in the prior 3 months, and 80% identified using a condom during sex every time in that same time span. In total, 61.8% reported lifetime use of illicit drugs, and of those that responded, 37.5% reported lifetime injection drug use. As well, 42.4% had been incarcerated. Only 18.2% reported making a round trip border crossing in the previous 6 months and 25% had ever been deported from the US to Mexico. Overall, this class can be characterized as heterosexual, low-income men and women with a high probability of illicit and injection drug use living and receiving HIV care in Mexico with limited cross-border mobility.

#### Class 5 (*n* = 33)

In this class, every member was recruited in Mexico, 97% had residence there the prior 6 months, 100% had health coverage there and none had health coverage in the US. The mean age was 45.97 years. In total, 69.7% were men and 45.5% of members identified as LGBT. This class had the lowest income at $32.94/week with 51.5% reporting having no income and 72.7% being unemployed. While 42.4% had not completed secondary school, 54.6% had completed high school. Every member reported having traded sex, members reported an average of 2.25 sexual partners in the prior 3 months, and 58.3% identified having used a condom every time during sex over the same time span. Every member had a lifetime history of illicit drug use and 30.3% reported having ever injected drugs. Members reported ever having been incarcerated at the highest proportion of any class at 81.8%. In all, only 3% had made a round trip border crossing in the previous 6 months and almost half (46.9%) had ever been deported from the US to Mexico. Overall, this class can be characterized as men and women, living and receiving care in Mexico, with history of illicit drug use, trading sex, deportation, and incarceration, with no cross-border mobility, and no or low income.

### Class Comparison of CAM Utilization and Attitudes/Beliefs

While 71.1% of all participants reported utilizing CAM in the prior 6 months, there were significant differences in usage rates between classes (*p* < 0.001; see Table 3). While 81.2, 88.2, and 90.9% of members of Classes 3, 4, and 5, respectively, reported using CAM, only 44.4% of Class 1 and 54.2% of Class 2 reported using CAM. While all classes utilized herbal/plant remedies and vitamin supplements at similar rates, there were significant differences in the utilization of both teas (*p* < 0.05) and body work (*p* < 0.001; i.e., acupuncture, chiropractors, and massage). Zero members of Class 1 identified using body work, whereas approximately 40% of both Classes 4 and 5 did so in the prior 6 months. As well, only 25% of Class 1 reported using teas, compared to 63.4, 73.3, and 71.0% of Classes 3, 4, and 5, respectively. There were almost no reports of accessing CAM practitioners, such as *curanderos, espiritistas*, or herbalists.

**Table 3:** CAM utilization and related attitudes and beliefs (by class).

	**Class**					
	**Total (*n* = 201)**	**1 (*n* = 27)**	**2 (*n* = 59)**	**3 (*n* = 48)**	**4 (*n* = 34)**	**5 (*n* = 33)**
**CAM USE, PAST 6 MONTHS**						
Any CAM^***^	143 (71.1%)^∧^	12 (44.4%)	32 (54.2%)	39 (81.2%)	30 (88.2%)	30 (90.9%)
Herbs/Plants	41 (27.9%)	2 (16.7%)	13 (39.4%)	12 (29.3%)	8 (26.7%)	6 (19.4%)
Teas^*^	87 (60.0%)	3 (25.0%)	14 (45.2%)	26 (63.4%)	22 (73.3%)	22 (71.0%)
Vitamins	87 (60.0%)	8 (66.7%)	17 (53.1%)	26 (65.0%)	18 (60.0%)	18 (58.1%)
Body Work^***^	47 (23.4%)	0 0.0%)	12 (20.3%)	8 (16.7%)	13 (38.2%)	14 (42.4%)
**CAM ATTITUDES AND BELIEFS**						
**Agrees with following statements:**						
“Prescription drugs should only be used as a last resort for illness”^**^	36 (17.9%)	3 (11.1%)	5 (8.5%)	7 (14.6%)	8 (23.5%)	13 (39.4%)
“I prefer natural remedies to prescription drugs”	24 (11.9%)	1 (3.7%)	7 (11.9%)	7 (14.6%)	7 (20.6%)	2 (6.1%)
“It's necessary to take medication doctors prescribe”	189 (94.0%)	25 (92.6%)	57 (96.6%)	44 (91.7%)	32 (94.1%)	31 (93.9%)
“[CAM] are important for maintaining good health”	146 (72.6%)	15 (55.6%)	42 (71.2%)	38 (81.2%)	26 (76.7%)	24 (72.7%)
“Using [CAM] allows me to have control over my health”^***^	81 (40.3%)	5 (18.5%)	19 (32.3%)	25 (52.1%)	23 (67.7%)	9 (27.3%)
“Prescription drugs are effective for illness”	171 (85.1%)	23 (85.2%)	47 (79.7%)	40 (83.3%)	31 (91.2%)	30 (90.9%)
“Using [CAM] allows me to hide my illness”	26 (12.9%)	1 (3.7%)	10 (16.9%)	3 (6.2%)	7 (20.6%)	4 (15.2%)
“[CAM] should only be used ^*^ for minor symptoms”^**^	92 (45.8%)	3 (11.1%)	22 (37.3%)	26 (54.2%)	23 (67.6%)	18 (54.5%)
**Reasons identified for using CAM:**						
Regular medical treatments do not help	39 (19.9%)	4 (15.4%)	9 (19.5%)	12 (25.0%)	6 (18.8%)	8 (25.0%)
Regular medical treatments too expensive^***^	97 (49.5%)	4 (15.4%)	18 (31.0%)	28 (58.3%)	21 (65.6%)	26 (81.2%)
CAM helps medical treatment^**^	61 (31.1%)	1 (3.8%)	11 (19.0%)	19 (39.6%)	13 (40.6%)	17 (53.1%)
Don't think that ART works	28 (14.3%)	5 (19.2%)	7 (12.1%)	7 (14.6%)	3 (9.4%)	6 (18.8%)
To treat ART side effects	79 (40.3%)	6 (23.1%)	17 (29.3%)	24 (50.0%)	16 (50.0%)	16 (50.0%)
Medical professional suggested them	37 (18.9%)	2 (7.7%)	13 (22.4%)	9 (18.8%)	8 (25.0%)	5 (15.6%)
Curiosity	53 (27.0%)	2 (7.7%)	14 (24.1%)	18 (37.5%)	7 (21.9%)	12 (37.5%)

* *p < 0.05*,

** *p = 0.001*,

*** p < 0.001, and

∧ *proportions based on total respondents to survey items*.

Nearly all participants believed that prescription medication is effective and that it is necessary to take the medications prescribed by a doctor, but at least 11% of every class stated that prescription drugs should only be taken as a last resort, with 23.5% of Class 4 and 39.4% of Class 5 believing such (*p* = 0.001). A majority of participants in all classes, ranging from 55.6% of Class 1 to 81.2% of Class 3, identified CAM as being an important part of maintaining good health and 11.9% of participants identified preferring CAM to prescription medication, ranging from only 3.7% of Class 1 to 20.6% of Class 4. Overall, 40.3% of participants identified that CAM can help them hide their HIV symptoms from others, but there was a significant difference between groups (*p* < 0.001); only 25–33% of participants in Classes 1, 2, and 5 agreed to such compared to 50% of Class 3 and 67% of Class 4.

### Class Comparison of HIV Care Engagement and Adherence

Overall, a majority (72.5%) of all participants identified as being in “good” or better health, 40.1% had been diagnosed with AIDS, and 89.9% were currently taking ART, with no significant difference amongst classes (see Table 4). In the prior month, 28.5% reported missing taking their ART at least once, 39.7% reported not always following the exact time schedule for their ART doses, and 17.8% reported not taking their ART medication for at least 1 weekend. However, there were notable differences between classes. Only 8.3% of Class 1 reported missing any ART in the prior month, whereas between 20.7 and 37.9% in the other classes reported doing so. The only significant difference identified was that while 0% of Class 1 identified not taking ART for at least 1 weekend in the same time span, over 20% of Classes 2 and 3 and over 30% of Class 5 identified as such (*p* < 0.05). Of participants who had been diagnosed with HIV at least 3 months prior to the interview, 18.5% identified having had at least one 3-month period not taking ART, with rates ranging from 9.8% of Class 3 to 29.1% of Class 2.

**Table 4:** ART adherence and health indicators (by class).

	**Class**					
	**Total (n = 201)**	**1 (*n* = 27)**	**2 (*n* = 59)**	**3 (*n* = 48)**	**4 (*n* = 34)**	**5 (*n* = 33)**
**Self-Rating of Overall Health**						
Excellent	46 (23.0%)∧	5 (18.3%)	11 (18.6%)	9 (18.8%)	12 (36.4%)	9 (27.3%)
Very Good	37 (18.5%)	6 (22.2%)	14 (23.7%)	7 (14.6%)	4 (12.2%)	6 (18.2%)
Good	62 (31.0%)	7 (25.9%)	19 (32.2%)	17 (35.4%)	8 (24.2%)	11 (33.3%)
Fair	7 (3.5%)	0 (0.0%)	2 (5.1%)	1 (2.1%)	0 (0.0%)	3 (9.1%)
Poor	46 (23.0%)	9 (33.3%)	11 (18.6%)	13 (27.1%)	9 (27.3%)	4 (12.1%)
Very Poor	2 (1.0%)	0 (0.0%)	1 (1.7%)	1 (2.1%)	0 (0.0%)	0 0.0%)
Diagnosed with AIDS	79 (40.1%)	13 (48.1%)	26 (44.8%)	14 (29.8%)	11 (33.3%)	15 (46.9%)
Currently Taking ART	178 (89.9%)	24 (92.3%)	55 (93.2%)	42 (87.5%)	28 (87.5%)	29 (87.9%)
**ART ADHERENCE**						
**In the prior 4 days:**						
Missed taking any ART	31 (17.2%)	5 (20.0%)	14 (25.5%)	9 (21.4%)	1 (3.4%)	2 (6.9%)
Didn't always follow ART schedule	58 (32.6%)	6 (25.0%)	23 (42.6%)	12 (28.6%)	6 (20.7%)	11 (37.9%)
Forgot to take ART previous weekend	12 (6.9%)	0 (0.0%)	3 (5.6%)	2 (4.8%)	4 (13.8%)	3 (12.0%)
**In the prior month:**						
Missed taking any ART	51 (28.5%)	2 (8.3%)	18 (32.7%)	14 (31.8%)	6 (20.7%)	11 (37.9%)
Didn't always follow ART schedule	71 (39.7%)	7 (29.2%)	24 (43.6%)	18 (42.9%)	10 (34.5%)	12 (41.4%)
Stopped taking ART during weekends^*^	32 (17.8%)	0 (0.0%)	12 (21.8%)	9 (22.0%)	2 (7.1%)	9 (31.0%)
Ever stopped taking ART for more than 3 months	33 (18.5%)	4 (16.0%)	16 (29.1%)	4 (9.8%)	4 (13.8%)	5 (17.9%)
Ever missed ART pickup, prior 6 months	18 (10.1%)	1 (4.0%)	6 (10.9%)	3 (7.1%)	2 (6.9%)	6 (21.4%)

* p < 0.05, ** p = 0.001, *** p < 0.001, and ∧ proportions based on total respondents to survey items.

## Discussion

Study findings reveal that the five distinct classes represent a highly heterogeneous group of Latino PLWHA, the profiles of which also indicate a spectrum of risk behaviors and relationship to the US-Mexico border region. Significant differences were noted between classes, including privileges associated with factors such as higher income and cross-border mobility, and barriers such as poverty, unemployment, engagement in sex work, and illicit substance use. Across all classes, CAM utilization was high and barriers to ART adherence were present, though study participants were largely well-engaged in HIV treatment.

The differences in CAM utilization by class appear to be correlated to which country individuals had access to care in, with Classes 1 and 2, the two classes with greater US health coverage, reporting less CAM utilization than the other classes. This relationship between country and CAM utilization could be indicative of cultural differences or of different environmental and structural factors in accessing CAM and HIV care in Tijuana and San Diego. Future research should focus on illuminating the nature of this relationship. Despite these significant differences in recent CAM utilization by class, the CAM utilization rates ranging from nearly 50% to over 90% indicate that CAM use is prevalent across all classes and is ubiquitous in some. These utilization rates are consistent with the prior literature (26, 30). It is thus clear that addressing CAM use must be a component of any HIV treatment modality for Latino PLWHA in the border region. Research into the interaction between CAM use and ART adherence is of immediate importance. We were unable to undertake such an analysis due to a lack of statistical power, but if a significant relationship between CAM use and ART adherence were to be found, this would provide valuable direction for research and interventions aimed at improving ART adherence amongst Latino PLWHA. As well, HIV care providers in the border region would benefit from increased CAM education opportunities and CAM-related protocols to empower them to continue providing effective care to their patients (32).

Given that Latino PLWHA are already at increased risk of being diagnosed with HIV later than non-Latino Whites and given the high rate of Latinos in San Diego County who progress to AIDS within a month of their HIV diagnosis (22), it is imperative that all classes of individuals living with HIV/AIDS be identified and that barriers to HIV care access remain an area of ongoing research. *An immediate research question is whether the classes identified here represent populations fully engaged in HIV care or if they represent a subset of a population in which many are not able to access care*. For example, it may be fair to hypothesize that Class 1, characterized by higher income, border mobility, and low risk profile, is reflective of a population in which all members are able to and do access HIV care. In comparison, it may be hypothesized that there is an un-sampled population with a similar risk profile as Class 5, characterized by high rates of illicit drug use, sex work, and incarceration, no income, and no border mobility, which is unable to access HIV care. While this research is only reflective of classes of individuals who have made contact with HIV-related services, the results provide a foundation for investigating and understanding the context of Latino PLWHA living in the border region who are not in contact with HIV care services. Research in identifying such populations is crucial in ensuring improved HIV-related health care engagement and outcomes for PLWHA Latinos in the border region.

As well, given that the US-Mexico border is at the forefront of the current North American political climate, it is important to reflect on how these results can inform how U.S. border policies may impact ability to access necessary care among Latino PLWHA living within the border region. The most important connection between this research and border policy implementation is that not all Latino PLWHA will be affected in the same way. Policy initiatives which seek to diminish or even stop border crossings, as the Trump Administration has recently called for (52), will disparately impact binational Latino PLWHA. Interestingly, though, new local binational insurance companies may mitigate the effects of federal policy aimed at decreasing border mobility. For example, SIMNSA and MediExcel are health insurance companies, that are approved by the State of California to provide health care services on both sides of the California-Mexico border (53). As a growing industry, binational insurance plans represent a promising solution for increasing access to care along the border, especially in the context of sister cities (such as San Diego and Tijuana) where crossing the border, for many, is a part of daily life. Future research should investigate the impact of U.S. border policies and bi-national insurance providers on access to care for the heterogeneous groups of Latino PLWHA living in the US-Mexico border region.

Finally, these findings indicate that, for future research and interventions directed toward Latino border populations, a theoretical foundation which recognizes the heterogeneity of the population and thus, which accurately reflects the population's narratives and needs, is necessary. Intersectionality provides such a framework and its relevance to addressing global health issues is gaining increased attention (54, 55). Intersectionality posits that individuals' experiences cannot be considered to be the causal result of a singular personal attribute, such as gender, race, or class, but instead, are the result of the interaction of all of these attributes with the many environmental contexts to which individuals are simultaneously subjected (52) Kapilashrami and Hankivsky discuss two strengths of applying intersectional theory to issues of global health which are of immediate relevance to the findings of this research: (1) that intersectional theory can be used to understand the heterogeneity of populations “that are often portrayed as relatively homogenous” [(54), p. 2,589]; and (2) that it can illuminate the many environmental contexts which shape individual and group experiences and thus, can provide a more accurate depiction of the source of health disparities (54). The findings presented here indicate that the population of Latinos seeking HIV care in the San Diego-Tijuana border region is heterogeneous; each group is subject to unique barriers to receiving care based on their geographic location, ability to cross the border, and class, amongst other factors. Intersectionality provides a helpful theoretical foundation for addressing health disparities faced by Latinos in the US-Mexico border region and should be considered in the design of future research and interventions to address them.

## Limitations

This study faces several limitations. First, in regard to the analysis itself, it is convenient to discuss “class membership” as though the five classes identified are distinct communities. It is important to understand that “class membership” does not necessarily refer to individuals belonging to a tangible group, but that individuals share a similar profile across a constellation of relevant variables. Next, the sampling method ensured that only individuals engaging with care and social services organizations were included in the study. Thus, the results are not generalizable to all Latino PLWHA in the San Diego-Tijuana region, but only to those in contact with such care organizations. It must be added, though, that the sampling method does not imply participants are necessarily currently receiving HIV care, as some of the recruitment service facilities did not directly provide care, but only helped connect individuals with care providers. In regard to measuring ART adherence, it would have been preferable to utilize a biological measure as opposed to self-report, though research indicates that self-report is a valuable tool for assessing ART adherence (56). As a cross-sectional study, participant circumstances that may underlie categorization in one category are not fixed, and temporally-sensitive factors, such as location of home or service access may change over time. Finally, given that the data is cross sectional, no causal inference can be made about any of the findings presented. Nevertheless, this study provides insights into diverse categories of Latino PLWHA in the U.S.-Mexico border region, and as such, can provide a framework from which to understand health needs and serve to inform service delivery models for diverse populations.

## Conclusion

This study establishes the heterogeneity of Latino PLWHA living in the border region and the widespread utilization of CAM across the population. The results of this study indicate the ability to access and adhere to care for Latino PLWHA is complicated by demographic factors, HIV risk profile, and binational mobility. The distinct experiences of this heterogeneous population must be illuminated to ensure the provision of culturally appropriate and necessary care to all individuals in need. As CAM use was prevalent (or ubiquitous) across all classes identified, incorporating CAM into HIV/AIDS care provision protocols is crucial for ensuring both effective and culturally relevant treatment. In addition, it is vital that classes not identified in this study, representing individuals not engaged with at any stage within the HIV continuum of care, be sought and understood to ensure that they are able to access needed care. Finally, it is crucial that future research utilize a guiding framework, such as intersectionality, which acknowledges the wide heterogeneity of Latino PLWHA in the US-Mexico border region.

## Ethics Statement

This research was conducted in accordance with the recommendations of the University of California, San Diego Human Research Protection Program, and the San Diego State University Human Subjects Institutional Review Board. All subjects gave written informed consent in accordance with the Declaration of Helsinki. The protocol was approved by the San Diego State University Human Subjects Institutional Review Board.

## Author Contributions

CM and MZ are responsible for all components of manuscript producing including the conceptualization, hypothesis formation, statistical analysis, and manuscript production. Both provide approval for the publication of its contents.

### Conflict of Interest Statement

The authors declare that the research was conducted in the absence of any commercial or financial relationships that could be construed as a potential conflict of interest. The reviewer CHS declared a shared affiliation, with no collaboration, with the authors to the handling editor at time of review.
